# Lidocaine Infusion: An Antiarrhythmic With Neurologic Toxicities

**DOI:** 10.7759/cureus.23310

**Published:** 2022-03-19

**Authors:** Yasmeen M Daraz, Omar H Abdelghffar

**Affiliations:** 1 Internal Medicine, Montefiore Medical Center, Bronx, USA; 2 Internal Medicine, Icahn School of Medicine at Mount Sinai, Elmhurst Hospital Center, Elmhurst, USA

**Keywords:** neurotoxic, seizure, ventricular tachycardia, antiarrhythmic, lidocaine infusion

## Abstract

As a renowned local anesthetic agent of choice, lidocaine is also a class 1b antiarrhythmic agent that is primarily used for the treatment of ventricular arrhythmias. Although lidocaine systemic toxicity is rare, it may be life-threatening; thus, its early identification and management are of vital importance. This case report details the clinical scenario of intravenous lidocaine administration to a patient at high risk of toxicity in a 64-year-old patient, who initially presented with sustained monomorphic ventricular tachycardia received lidocaine and subsequently developed neurologic manifestations of lidocaine toxicity, including altered mental status and seizure. The patient was treated promptly with benzodiazepine and discontinuation of lidocaine as the offending agent, with complete resolution of adverse effects.

## Introduction

Lidocaine is a common local and systemic anesthetic that is widely used in primary care offices, emergency departments, and operating rooms. Its rapid onset of action is appropriate and desirable in certain settings, but this characteristic can also be deadly, with multiple reports of cardiac arrest, seizure, and other neurologic symptoms [1]. These neurologic symptoms include insomnia or drowsiness, light-headedness, dysarthria, change in mental status, and personality changes. In addition, high plasma concentrations of lidocaine can increase neuronal excitability and provoke seizures, which are usually generalized [1]. These side effects may be particularly frequent in elderly patients, those with heart failure, or those with significant liver dysfunction in whom the metabolism of lidocaine is reduced. Treatment of lidocaine toxicity is primarily supportive but should include airway protection for hypoxia, benzodiazepines for seizure activity, and cardiac pacing or atropine for bradycardia. Because lidocaine is 70% metabolized in the liver, it is important to maintain vigilance for concomitant drugs that act upon the cytochrome P450 (CYP 450) pathway [2]. Monitoring for early detection of subtle neurologic adverse effects is essential to avoid symptom progression, as elucidated herein. 

## Case presentation

A 64-year-old man with an implantable cardioverter-defibrillator (ICD) presented to the emergency department after receiving seven ICD shocks at home. His medical history was significant for isolated cardiac sarcoidosis, currently treated with steroids, previous left heart catheterization with mild nonobstructive coronary artery disease, ventricular tachycardia/ventricular fibrillation (VT/VF) with ICD placement, which was previously treated with amiodarone but since been discontinued because of acquired hyperthyroidism, currently treated with methimazole, heart failure with a reduced ejection fraction of 15% recovered to 45%, cerebral aneurysm with clipping, provoked pulmonary embolism for which he has completed three months of anticoagulation, and chronic kidney disease stage 3.

**Figure 1:**
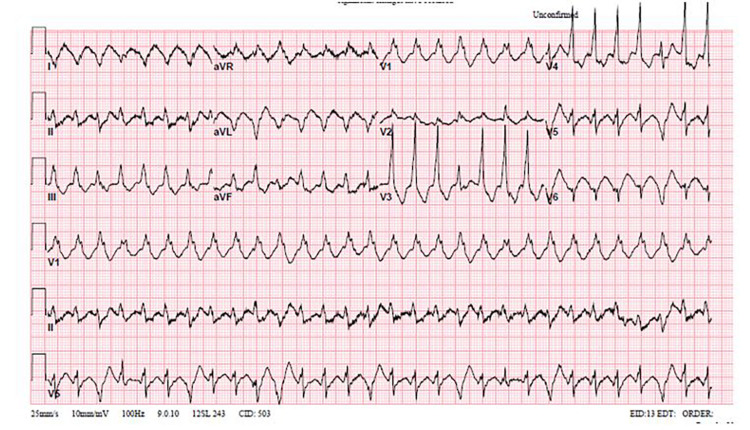
Ventricular fibrillation

**Figure 2:**
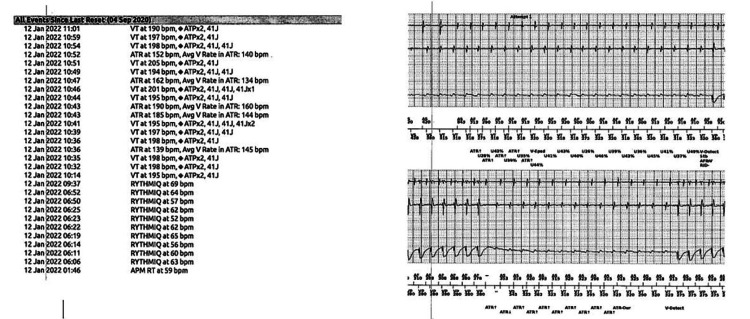
Implantable cardioverter-defibrillator interrogation findings

Initial vital signs on presentation to the emergency department were blood pressure 155/83 mm Hg, heart rate 138 beats per minute, temperature 98.3°F, respiratory rate 22 breaths/minute, and oxygen saturation 98% on room air. Pertinent physical examination findings were chest pain and tachycardia. He received one ICD shock during the physical examination. He was found to have multiple episodes of sustained monomorphic VT with ICD shocks that did not convert to normal sinus rhythm, suggesting the patient was in a VT storm (Figure 1). An electrophysiology consultation was sought for device interrogation, which found multiple episodes of VT at about 190 beats per minute and multiple cycle lengths with ICD anti-tachycardia pacing/shocks not converting to normal sinus rhythm, as well as 35% atrial pacing and 3% ventricular pacing (Figure 2). Normal sinus rhythm was restored with intravenous lidocaine and amiodarone bolus with a drip. Laboratory studies showed electrolytes, thyroid function, liver function, and urine drug screen results within normal limits. In addition, the patient was incidentally found to be positive for coronavirus disease 2019 (COVID-19).

For further monitoring, the patient was admitted to the cardiac intensive care unit. Lidocaine drip was continued at 1 mg/min, and mexiletine 200 mg orally every eight hours was initiated. Lidocaine infusion was weaned on hospital day two to 0.5 mg/min to bridge with mexiletine 200 mg orally every eight hours. During hospital day two, the patient was observed to have intermittent confusion with a waxing/waning pattern, which prompted the team to terminate the lidocaine infusion. The patient remained stable after cessation of lidocaine until that night when he had an episode of unresponsiveness with eyes open but an inability to follow commands, associated with drooling and shaking of his jaw, which was a significant deviation from his baseline status of full alertness and orientation. Telemetry showed a heart rate of 130-140 beats per minute with a poor baseline. He quickly recovered one minute after the episode without intervention and began to respond to the examiner’s questioning. He was evaluated by the neurology team, and it was agreed that the patient was post-ictal secondary to what was most likely a seizure caused by lidocaine toxicity. The patient had no previous seizure history. Both the lidocaine and mexiletine were stopped, and 4 mg of lorazepam were administered. Because of the life-threatening nature of arrhythmia and seizures, amiodarone was restarted at 400 mg/day for seven days, followed by 200 mg/day, despite the previous adverse thyroid effects. Computed tomography of the head was unremarkable with noted chronic post-procedural changes in the right anterior temporal lobe and a middle cerebral artery coil. Although the intracranial abnormality of a right middle cerebral artery aneurysm and the clip was considered to be a potential nidus for seizures, he had no history of seizures, so the team determined that initiation of antiepileptics was not needed. Electroencephalography was deferred because of the expected abnormalities that would not change current management.

Transthoracic echocardiography was performed, which showed a reduced ejection fraction of 35% from 45% and moderate diffuse left ventricular hypokinesis with normal right ventricular function. Given the reduced ejection fraction and episode of VT storm, there was a concern for an acute sarcoid flare, so the patient was started on 40 mg of oral prednisone daily. Of note, oral steroids were started after the seizure-like episode mentioned above.

On day five, the patient was electrically stable and without further episodes of VT in the last 24 hours before discharge. Before discharge, adjustments to the ICD thresholds were performed. Discharge plans for follow-up included cardiac magnetic resonance imaging to assess endocardial scarring, in addition to 18F-fluorodeoxyglucose positron emission tomography to plan for VT ablation. Because the patient was positive for COVID-19, these assessments were deferred to the outpatient setting. The medication and discharge plan included amiodarone 400 mg thrice daily for three more days, followed by amiodarone 200 mg/day, metoprolol 50 mg twice daily, prednisone 40 mg/day, aspirin 81 mg/day, atorvastatin 40 mg/day, and sacubitril/valsartan 24-26 mg/day, in addition to cardiology/electrophysiology follow-up.

## Discussion

Lidocaine is a common drug used in multiple specialties that can be administered either by injection or inhalation or as a topical agent. Its most common uses are based on its benefit as a general amide-type local anesthetic and as an antiarrhythmic agent to depress ventricular arrhythmias. Lidocaine is classified as a class Ib antiarrhythmic that decreases the permeability of the neuron membrane to sodium, which causes inhibition of depolarization, resulting in blocked conduction. The main body systems targeted by lidocaine are the cardiovascular system and central nervous system. Because the central nervous system has heightened sensitivity to electrophysiologic changes, neurologic symptoms precede those of the cardiovascular system. These central nervous system effects can manifest as agitation, anxiety, confusion, lethargy, loss of consciousness, paresthesia, psychosis, seizures, and slurred speech [3]. Adverse reactions from lidocaine exert dose-dependent effects on the central nervous system. In particular, lidocaine has a dose-dependent anti-epileptic effect, with serum concentrations less than 5 mg/mL decreasing neuronal excitability; therefore, its use may be implicated in the treatment of status epilepticus [4]. In contrast, at higher concentrations, lidocaine is a proconvulsant that lowers the seizure threshold and induces seizures. Although the specific biochemical mechanism is not completely understood, it has been noted that supratherapeutic concentrations of lidocaine block inhibitory cortical neurons that lead to convulsions [5]. Factors such as dose, speed of administration, presence of disease, and patient age and sex play a role in the predisposition to lidocaine toxicity.

As seen in this case, the diagnosis of lidocaine toxicity is typically clinical, and serum levels are not the standard of care to determine lidocaine toxicity. Despite the dose of lidocaine infusion being within normal limits, lidocaine toxicity is an entity dependent on various patient-related risk factors, namely electrolyte derangements and liver function test abnormalities. As evident in this case report, although the lidocaine infusion dose may have been within the normal range, it is critical to note that the patient was simultaneously receiving lidocaine and mexiletine, both of which are metabolized by the CYP 450 pathway in the liver, with mexiletine being a moderate inhibitor of CYP 1A2, which may have been a contributing factor to the adverse effects experienced by this patient [6]. In addition, although convulsions in response to lidocaine infusion are uncommon, the medical care team should be educated about how to manage these adverse effects-no matter how benign a medication can appear. Although the patient, in this case, recovered spontaneously once lidocaine infusion was stopped, the American Society of Regional Anesthesia and Pain Medicine recommended a bolus of 1.5 mL/kg of 20% intravenous lipids followed by continuous infusion for management of cardiac instability [7].

## Conclusions

Despite the daily use of lidocaine in many practice settings, an understanding of the importance of its potential toxicities and knowledge of how to manage them is vital. These toxicities can manifest as life-threatening conditions that may initially present as subtle neurological symptoms but can progress quickly to overt status epilepticus. Identifying and treating lidocaine toxicity that presents with status epilepticus with early benzodiazepines can prevent long-term neurologic sequelae.
